# Uptake of potentially toxic elements by edible plants in experimental mining Technosols: preliminary assessment

**DOI:** 10.1007/s10653-021-01091-x

**Published:** 2021-10-21

**Authors:** María José Martínez-Sánchez, Carmen Pérez-Sirvent, Salvadora Martínez-Lopez, Mari Luz García-Lorenzo, Ines Agudo, Lucia Belen Martínez-Martínez, Carmen Hernández-Pérez, Jaume Bech

**Affiliations:** 1 Department of Agricultural Chemistry, Geology and Pedology, International Excellence Campus “Mare Nostrum”, University of Murcia, Murcia, Spain; 2 Department of Petrology and Geochemistry, Faculty of Geology, Moncloa Campus of International Excellence, Complutense University of Madrid, Madrid, Spain; 3 University of Barcelona, Barcelona, Spain

**Keywords:** Plant uptake, Metal(loid)s, Arsenic, Technosols, Soil remediation

## Abstract

A study was carried out to evaluate the absorption of potentially toxic elements from mining Technosols by three types of vegetable plants (broccoli (*Brassica oleracea var. italica*), lettuce *(Lactuca sativa*) and onion (*Allium cepa*)), the different parts of which are intended for human and farm animal consumption (leaves, roots, edible parts). The preliminary results obtained highlight the importance of the design of the mining Technosols used for agricultural purposes, obtained from soils and sediments of mining origin and amended with residues of high calcium carbonate concentrations (limestone filler and construction and demolition wastes). The experiment was carried out in a greenhouse, and the total metal(loid)s concentration (As, Pb, Cd, Cu, Fe, Mn and Zn) of the soil, rhizosphere, aqueous leachates and plant samples was monitored, the translocation and bioconcentration factors (TF and BCF, respectively) being calculated. The characterization of the soils included a mobilization study in media simulating different environmental conditions that can affect these soils and predicting the differences in behavior of each Technosol. The results obtained showed that the levels of potentially toxic elements present in the cultivated species are within the range of values mentioned in the literature when they were cultivated in soils with calcareous amendments. However, when the plants were grown in contaminated soils, the potentially toxic elements levels varied greatly according to the species, being higher in onions than in lettuce. Experiments with the use of lime filler or construction and demolition wastes for soil remediation result in crops that, in principle, do not present health risks and are similar in development to those grown on non-contaminated soil.

## Introduction

Certain areas of the Mediterranean region, that were dedicated to mining in the past, today support agricultural activities of different degrees of intensity. However, doubts arise about the degree of contamination that may have affected the soil and any possible effect on crops. In many soils, the influence is residual, only manifesting itself as a geogenic increase in potentially toxic elements (PTEs) concentrations (García-Lorenzo et al., 2012), which do not affect crops or other activities that may develop in these areas. However, some soils may have been more affected than others and require recovery measures. One of the solutions proposed to respond to this problem is the construction of Technosols, as a better alternative to other traditional waste management practices, such as uncontrolled or even controlled discharge, inactivation or incineration processes, and valorization or recovery of useful materials (Camps et al., 2008). Technosols are a new Reference Soil Group, from the World Reference Base for Soil Resources (IUSS Work Group WRB, 2006), of artificial origin, formed by mixtures of different wastes and non-hazardous by-products (Camps et al., 2008; Sayadi-Gmada et al., 2019). The main applications of Technosols are in agriculture, the recovery of degraded and/or contaminated soils, landfill coverage, and in areas affected by urban construction or infrastructure projects (Echeverria and Morel, 2015; Grard et al., 2020).

Technosols may be considered a solution for the recovery/restoration of mines and quarries or soils degraded by erosion, fire or the loss of productive capacity. The development of mixtures to obtain these artificial soils has a dual purpose; on the one hand, waste is valorized, minimizing the possible environmental impact arising from their mismanagement and, on the other hand, degraded soils can be recovered without excessive cost (Aguilar et al., 2018; Ruiz et al., 2020; Slukovskaya et al., 2019). In this way, it is possible to avoid the unwanted and unnecessary disposal of many types of waste and currently underused products, prolonging their useful life, while meeting environmental objectives and favoring the fight against climate change (Ferronato and Torretta 2019: UNEP, 2015).

The soils produced from these materials are intended to improve soil properties and reduce the transfer of PTEs to groundwater. For this purpose, organic waste from the wood, leather and textile industries and even sewage sludge have been used (Egiarte et al., 2008; Wang et al., 2008), as composts of agricultural waste (Clemente et al. 2012), industrial waste (Calace et al., 2005; Monserie et al., 2009), residues of animal origin, alone or mixed with other organic wastes (Clemente et al., 2012; De la Fuente et al., 2011; Kabas et al., 2012; Lwin et al, 2018; Pardo et al., 2014). Other wastes that have been used include demolition and construction wastes (DCWs), quarry waste, mine waste, fly ash, limestone materials and clays, alone or combined, marble sludge blends and compost (Lee et al., 2009; Melgar-Ramírez et al., 2012; Santos et al., 2019; Wang et al., 2014) and sepiolite (Abad-Valle et al., 2016).

The use of limestone filler (LF), residue of limestone quarries, as an amendment for the recovery of soils affected by mining activities is an eco-efficient solution for revaluing a residue and neutralizing the effects of the supergenic alteration of iron sulfides present in these soils (Martínez-Sánchez et al., 2014; Pérez-Sirvent et al., 2007, 2010). The use of DCWs is another option, since the high calcium carbonate content and alkaline pH of this by-product provide the same advantages as limestone filler.

The traceability of PTEs, to protect both human health and whole ecosystems, is one of the pressing tasks of researchers in the multidisciplinary field of risk analysis (RA). Therefore, another important aspect is to check the efficiency of Technosols, ensuring that they meet the objective of mining soil restoration, avoiding the mobility of PTEs and providing a substrate compatible with the development of crops that will not induce the transfer of PTEs to the trophic chain (Garau et al., 2021).

To carry out a comparative study of the uptake of PTEs by plants intended for human consumption in the different types of Technosols, it is necessary to monitor leachates, the plants themselves, the substrate and the crop type.

This work presents only preliminary results and focuses on the importance of the design of the mining Technosols suitable for agricultural purposes, since the experiments carried out were numerous, and the results are still in study.

## Materials and methods

### Materials: experimental design

To check the mobility and transfer of PTEs to plant species, a greenhouse experiment was designed using a material of mining origin that was representative of the mine soils in a particular area. For this, three sites of different mineralogical characteristics were chosen within the Sierra Minera de Cartagena-la Unión (SE Spain), grouping the different situations that have occurred in the area, mining waste in situ, mining waste transported by the watercourses (Ramblas) and forming sedimentation deltas on the seashore and waste dumped directly into the sea and filling up the beach.

The characteristics of these sites where the samples were taken are:

*S1* Portman Bay site: a bay full of sediments from Pb and Zn hydrometallurgy discharges into the sea and has been subjected to processes of supergenic alteration (Pérez-Sirvent et al., 2018).

*S2* Cabezo Rajao site: abandoned metal mining area (Pb and Zn) (Navarro et al., 2012).

*S3* Lo Poyo site: wetland zone near the Mar Menor lagoon and affected by the materials of mining origin that were transported by the Ramblas (Martínez-López et al., 2019).

**Fig. 1:**
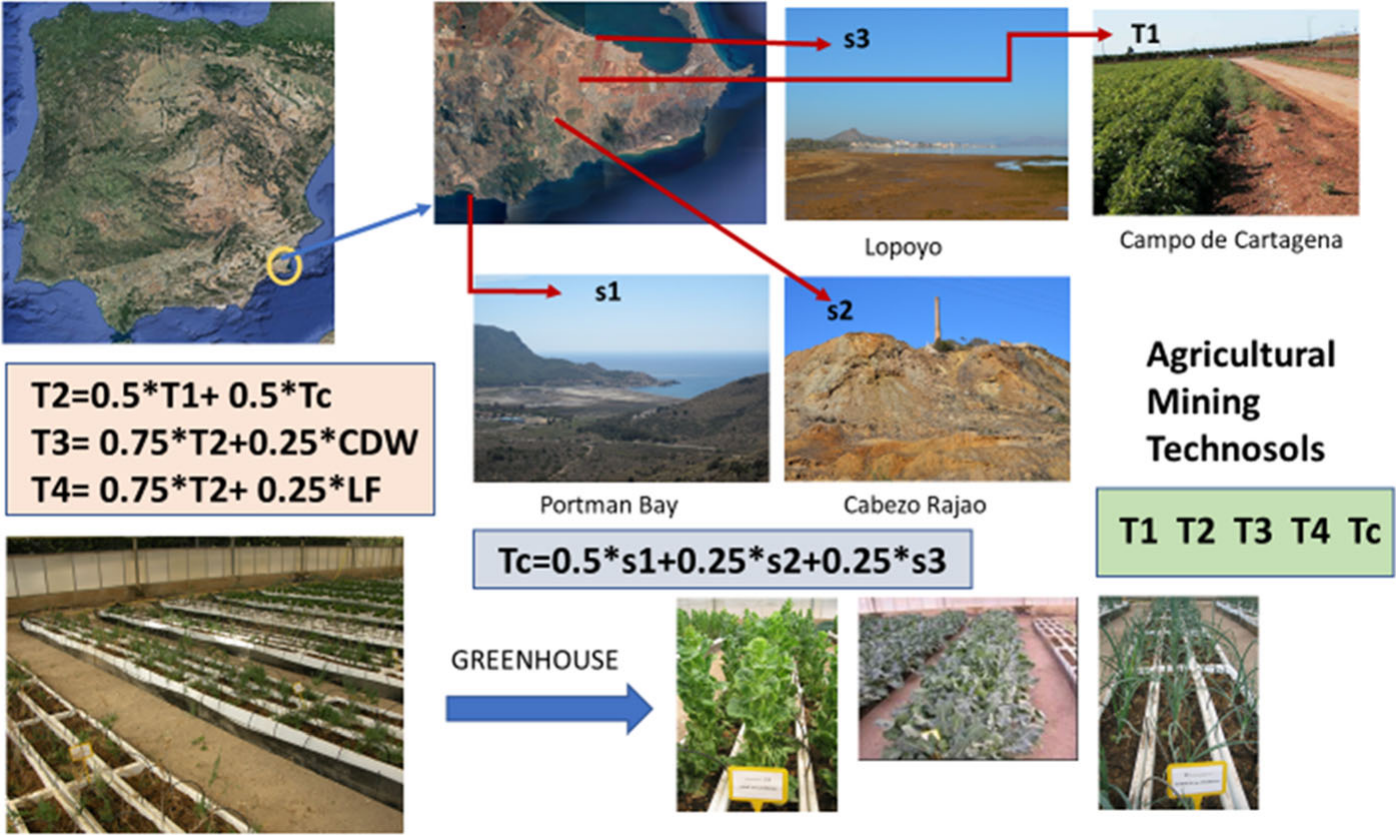
Location of the study area. Graphical summary of the way of preparation of the Technosols

The location of each of the sites as well as the extent of the affected mining area is shown in Fig. 1.

Amendments: A number of materials were used to manufacture the Technosols and check the performance, including local agricultural soils, LF and CDWs. The characteristics of these materials are given below:

- *Agricultural soils (T1)* Characteristic soils of the Campo de Cartagena are dedicated to agricultural use but not affected by mining. Soil samples were taken from close to the selected sites and used as control in the experiment.
- *Construction and demolition wastes (CDWs)* Provided by an inert waste treatment plant in the Region of Murcia.
- *Limestone filler (LF)* From an aggregate sorting plant of the Region of Murcia, near the facilities where the experiment was carried out.

The Technosols prepared for this experiment were as follows: Technosol TC was prepared from a mixture of three mining soils. For this purpose, two tons of soil from each selected site was collected using a small excavator and three large samples were mixed in an industrial mixer in order to obtain a single homogeneous material in the following proportions: Portman Bay (S1) (50%), Cabezo Rajao (S2) (25%) and Lo Poyo (S3) (25%). The result was a soil with a loam texture and with a very varied mineralogy and a high PTE concentration. It was considered as representative of the mining soils of the area, so that the results could be reasonably extrapolated to other soils of the area and representing the worst conditions for plant growth. Technosol T1 was made with a local agricultural soil, used as control for the experiment. Technosol T2 was made up as a mixture of Technosol TC and local agricultural soil, and Technosol T3 and T4 were made up as a mixture of T2 with CDW and LF, respectively (Table 1).

**Table 1:** Composition (%) of the experimental Technosol mixtures used

Technosols	S1	S2	S3	Agricultural soil	CDW	LF
T1	–	–	–	100.00	–	–
T2	25.00	12.50	12.50	50.00	–	–
T3	18.75	9.37	9.37	37.50	25.00	–
T4	18.75	9.37	9.37	37.50	–	25.00
TC	50.00	25.00	25.00	–	–	–

For this work, three widely consumed plant species cultivated in soils devoted to agriculture near the study area were selected:

- Family Asteraceae:
  - Baby lettuce var. Little Gem *(Lactuca sativa, L. var. capitata)*
- Family Brassicaceae:
  - Broccoli *(Brassica oleracea var. Italica)*
- Family Liliaceae
  - Onion variety Babosa *(Allium cepa L.)*

Soil mixtures were placed in pots. Each pot measured 1100 × 240 × 220 mm and had a useful capacity of 35 L. This made a total of 10 units of 21 pots, which were installed inside a greenhouse with lateral and overhead ventilation. The experiment was carried out in duplicate, with two test units for each Technosol. A total of 70 plants were sown for each cultivated species.

Irrigated herbaceous crops were grown in a greenhouse with fertigation, thus allowing environmental variables to be controlled. For leachates monitoring, ten 80-L capacity leachate collection tanks were used. Each tank received the leachate from three rows of seven expanded polystyrene pots filled with the soils being tested. The experiment was carried out in the facilities of the Murcia Institute of Agricultural and Food Research and Development (IMIDA). The duration of the experiment depended on the vegetative cycle of each crop, being 5 weeks for lettuce and 8 weeks for broccoli and onion, respectively.

### Methods

#### Sampling

A representative sampling of the materials used for the manufacture of the Technosols and a random sampling representative of each test unit (10–15 samples) of soil (rhizosphere) and plant was carried out. The leachates were collected in specially designed tanks at one end of each test subunit for each soil type and plant, as indicated above.

#### Analytical determinations

The samples were air-dried and sieved to < 2 mm for general analytical determinations. The pH and electrical conductivity were determined in (1:1, w/v) soil–water mixtures after shaking for 2 h, using pure water. Equivalent calcium carbonate (%) was determined by the classical volumetric method using a Bernard calcimeter previously calibrated against Na_2_CO_3_ (Hulseman, 1996). The active carbonate content was determined using a 0.1 M ammonium oxalate solution (Loeppert and Suárez, 1996). The organic carbon content was determined by sulfochromic oxidation (Nelson & Sommers, 1982), according to the NF-X31-109 standard (AFNOR, 1993).

Particle size distribution was evaluated by using a static light scattering (LS) instrument equipped with a microliquid module (Coulter LS 13,320, Beckman Coulter, Inc., Fullerton, CA, USA). The Coulter LS 13,320 software uses Mie theory to produce an optimal analysis of the light energy distribution and to obtain the size distribution of the particles.

Four selective chemical extraction methods were used to assess three relevant PTEs (As, Cd and Pb) and micronutrients (Zn, Cu, Fe and Mn) availability in soil, each of these simulates specific environmental conditions (Garcia-Lorenzo et al., 2014):

- Water medium: The procedure described in European Standard EN 12,457–1 (2002) was used, and the extracts were obtained from 1:5 (w/v) soil–water mixtures after shaking for 24 h, using distilled water. The extracts obtained by filtering through a 0.45-μm cellulose acetate disk filter were then analyzed.
- Nitric acid medium (Martínez Sánchez et al. 2011): 1 g of sample in 50 ml 0.1 M HNO_3_ and 1 hour of stirring, followed by centrifugation at 3000 rpm for 20 min.
- Complexing–reducing medium (Mehra & Jackson, 1960): 1 g of sample in 40 ml of sodium citrate 0.3 M and 5 ml of NaHCO_3_ 1 M. The suspension was heated to 80 °C in a water bath and 1 g of sodium dithionite was added; the suspension was stirred continuously for 1 min and discontinuously for 15 min. It was then centrifuged at 3000 rpm for 10 min.
- Oxidizing medium (step 3 BCR (Community Bureau of Reference)) (Sutherland & Tack, 2002): Forty milliliters of H_2_O_2_ (pH 2–3) was added to 1 g of sample. The mixture was maintained at room temperature for 1 h, after which the digestion continued in a bath at 85 °C until the volume was reduced to a few milliliters. Next, 10 ml of H_2_O_2_ (pH 2–3) was added again and the previous procedure was repeated. Subsequently, 50 ml of NH_4_OAc 1 M adjusted to (pH 2) was added and stirred for 16 h at 22 ± 5 °C and centrifuged at 3000 rpm for 20 min.

To determine the total PTE concentration, the soil samples were first ground to a fine powder using an agate ball mill. Samples were placed in Teflon vessels and 5 ml of concentrated HF acid solution, 2 ml of concentrated HNO_3_ acid solution and 5 ml of distilled water were added. When digestion in the microwave system was complete, the samples were transferred to a volumetric flask and brought to 50 ml before measurement. Teflon or other suitable plasticware was used for handling these liquids.

In the case of plants, fresh material was separated into roots and aboveground biomass, carefully washed with water to remove soil and dust particles, air-dried and then lyophilized. Then, 200 mg of lyophilized vegetal tissue was placed in Teflon vessels with 3 ml of water, 2 ml of concentrated H_2_O_2_ and 5 ml of concentrated HNO_3_ acid solution, and subjected to digestion, finally obtaining 50 ml solutions.

The soils and plant samples were digested using a Milestone ETHOS Plus Microwave system operating with a standard program. The reliability of the results was assessed through analysis of the NIST standard reference materials: SRM 2711 Montana Soil and SRM 1515 Apple leaves. Spikes, duplicates and reagent blanks were also used as a part of the quality control.

The As concentration was determined using atomic fluorescence spectrometry with an automated continuous flow hydride generation (HG-AFS) spectrometer (PSA Millennium Excalibur 10,055). Zinc, Fe and Mn concentrations were determined by flame atomic absorption spectrometry (FAAS) and Cu, Cd and Pb by electrothermal atomic absorption (ETAAS) using a PerkinElmer 800 atomic absorption spectrophotometer.

From the results obtained for plant and rhizosphere, the corresponding transfer factor (TF) and bioconcentration factor (BCF) have been calculated for each plant where transfer factor (TF) = PTE plant concentration/PTE soil concentration and bioconcentration factor (BCF) = PTE plant concentration/PTE root concentration.

A semiquantitative estimation of the mineralogical composition of the samples was made by X-ray diffraction (XRD) analysis using Cu-Kα radiation with a PW3040 Philips Diffractometer. The X-ray diffraction diagrams obtained by the crystalline powder method were analyzed using X-powder software (Martin, 2004). The powder diffraction file (PDF2) database was used for peak identification, considering the fact that the determination of minerals from soils by XRD analysis is not accurate below a limit of 5% of the total weight in a sample (depending on the crystallography of individual minerals).

All the statistical analysis of the experimental data was carried out using specialized software (IBM SPSS Statistics v-20).

## Results and discussion

### General characteristics of the soils

Table 2 presents the statistical summary of the characteristics of the soils and amendments used. The results were homogeneous for T1, T2, T3 and T4 Technosols, with a basic pH (7.90–8.50) and average calcium carbonate content. Electrical conductivity values were also similar, ranging from 4.91 to 6.39 mS cm^−1^. Calcium carbonate mean values ranged from 44.6% in T1 (followed by T4 and T3) to 25.4% in T2. The organic matter content was similar (0.6–1.4%) which would be due to the fertilizer used for cultivation. The TC soil had different characteristics from the Technosols, with an acid pH (4.6), high EC (22.9 mS cm^−1^) and no detected calcium carbonate or organic matter.

**Table 2:** Statistical summary of the characteristics of the Technosols (T1, T2, T3, T4 and TC) used in each crop and amendments (LF and DCW)

Samples	Statistical summary	pH	OM (g kg^−1^)	EC (mS/cm)	CaCO_3_ (g kg^−1^)	Active CaCO_3_ (g kg^−1^)	< 2 (µm) (g kg^−1^)	2–50 (µm) (g kg^−1^)	50–2000 (µm) (g kg^−1^)
T1	Range	7.92–7.99	12–16	5.10–5.40	430–460	24.2	48.5	622.5	330
	Median ± standard deviation	7.97 ± 0.03	14 ± 1.7	5.23 ± 0.13	450 ± 10.8				
T2	Range	7.69–7.86	13–14	6.37–6.45	222–278	12.9	75.1	405.9	520
	Median ± standard deviation	7.80 ± 0.08	13.8 ± 0.5	6.39 ± 0.04	254 ± 23.7				
T3	Range	7.90–7.95	11–12	4.90–4.93	296–366	14.2	97	623.6	280
	Median ± standard deviation	7.92 ± 0.02	11 ± 0.5	4.91 ± 0.01	34.2 ± 32.8				
T4	Range	8.49–8.51	5–6	5.09–5.18	352–421	18.7	88	437	480
	Median ± standard deviation	8.50 ± 0.01	6 ± 0.5	5.13 ± 0.04	392 ± 18.2				
TC	Range	4.50–4.79	< 2	22.7–23.3	–	–	12.4	151.6	840
	Median ± standard deviation	4.64 ± 0.12	< 2	22.9 ± 0.30	–	–			
LC		–	–	–	–	–	208.8	712	80
DCW		–	–	–	–	–	401.1	557	40

The results obtained for the particle size distribution of the starting materials showed that most samples had a loamy texture, since the 2–20 µm fraction predominated, while the filler had a finer texture (loamy clay).

The PTE concentration was high in Technosol TC, which corresponds to a mixture of mining materials, and very low in Technosol T1, the agricultural soil used as a control for the experiment (Table 3). Technosols T2, T3 and T4 showed intermediate contents between T1 and TC.

**Table 3:** Statistical summary of the PTE contents of the Technosols (T1, T2, T3, T4 and TC) used in each crop

Samples	Statistical summary	Zn (mg kg^−1^)	Pb (mg kg^−1^)	Cu (mg kg^−1^)	Cd (mg kg^−1^)	As (mg kg^−1^)	Fe (g kg^−1^)	Mn (mg kg^−1^)
T1	Range	95–115	< QL	16–18	0.1–0.2	4–6	14.30–18.30	279–290
	Median ± standard deviation	101.0 ± 12.89	(0.01)	17.0 ± 0.88	0.14 ± 0.1	5.5 ± 0.58	15.5 ± 0.91	285.0 ± 4.50
T2	Range	5489–6122	1520–1787	86–98	16–26	98–125	10.01–11.08	3629–3871
	Median ± standard deviation	5894.0 ± 78.91	1640.0 ± 96.71	93.0 ± 5.1	22.0 ± 1.31	119.0 ± 12.0	10.79 ± 0.054	3770.0 ± 104.0
T3	Range	4122–4588	956–1238	47–59	9.6–15	69–81	5.99–7.01	1779–1791
	Median ± standard deviation	4393.0 ± 55.92	1170.0 ± 88.97	52.0 ± 3.14	12.0 ± 1.77	75.0 ± 3.95	6.73 ± 0.064	1785.0 ± 4.50
T4	Range	3996–4113	900–1129	45–67	10–16	68–89	4.89–6.06	1555–1586
	Median ± standard deviation	4073.0 ± 89.12	990.0 ± 77.36	57 ± 9	13.0 ± 2.11	80.0 ± 4.88	5.31 ± 0.077	1570.0 ± 13.0
TC	Range	12,156–13,004	2577–2988	100–135	35–46	401–486	13.88–15.56	8292–8855
	Median ± standard deviation	12,782.0 ± 99.11	2745.0 ± 79.95	122.0 ± 4.88	40.0 ± 2.12	439.0 ± 21.0	14.62 ± 0.090	8570.0 ± 230.0

< QL = 0.01; Dry weight

The highest concentration of PTE corresponded to Zn, followed by Pb and As, which is in line with the areas of polymetallic Pb and Zn (galena and sphalerite) mineralization. Table 4 summarizes the mineralogical characteristics of the studied samples. Phyllosilicates were the most abundant at 1.0 nm. The amendments were composed of calcite, dolomite (> 60%) and phyllosilicates. The agricultural soil, T1, contained mostly calcite, followed by illite (phyllosilicate 1.0 nm) and quartz as the most representative minerals. The Technosol formed by the mixture of mine materials, TC, showed a mineralogical composition similar to that of soils of mining origin that were subjected to processes of supergene alteration, with phyllosilicates 1.4 and 1.0 nm (clinochlore and mica), jarosite, oxyhydroxides (akaganeite and goethite), among others. These minerals were also present in T2, T3 and T4 along with those provided by the amendments (DCWs and LF) and those of T1.

**Table 4:** Mineralogical composition of the Technosols (T1, T2, T3, T4 and TC) and amendments used (LF and DCW)

%	Phyl. 1.4 nm	Phyl. 1.0 nm	Quartz	Albite	Calcite	Dolomite	Siderite	Akaganeite	Hematite	Gypsum	Jarosite
T1	4	32	12	2	34	10	–	–	2	4	–
T2	6	35	12	1	20	5	6	–	–	5	10
T3	5	38	13	1	30	5	–	–	2	6	–
T4	3	20	10	1	35	20	–	–	2	6	–
TC	14	25	9	2	–	–	12	10	2	6	20
DCW	12*	30	8		40	–	–	–	–	10	–
LF	–	3	4		60	33	–	–	–	–	–

^*^Tobermorite

The high conductivity points to the presence of soluble salts which were not identified in this case. These conductivity values are not justified by the presence of gypsum in the samples, so the soluble salts must be at concentrations below the XRD quantification level.

#### Potential toxic elements mobility

To be able to predict the behavior of Technosols and to ascertain any differences among them, a mobilization study was carried out in different media—water (W), acid (A), a complexing–reducing medium (Mhera-Jackson, MJ) and oxidizing environment (Ox)—in order to simulate different environmental conditions that may affect these soils (Martínez Sánchez et al. 2011).

**Fig. 2:**
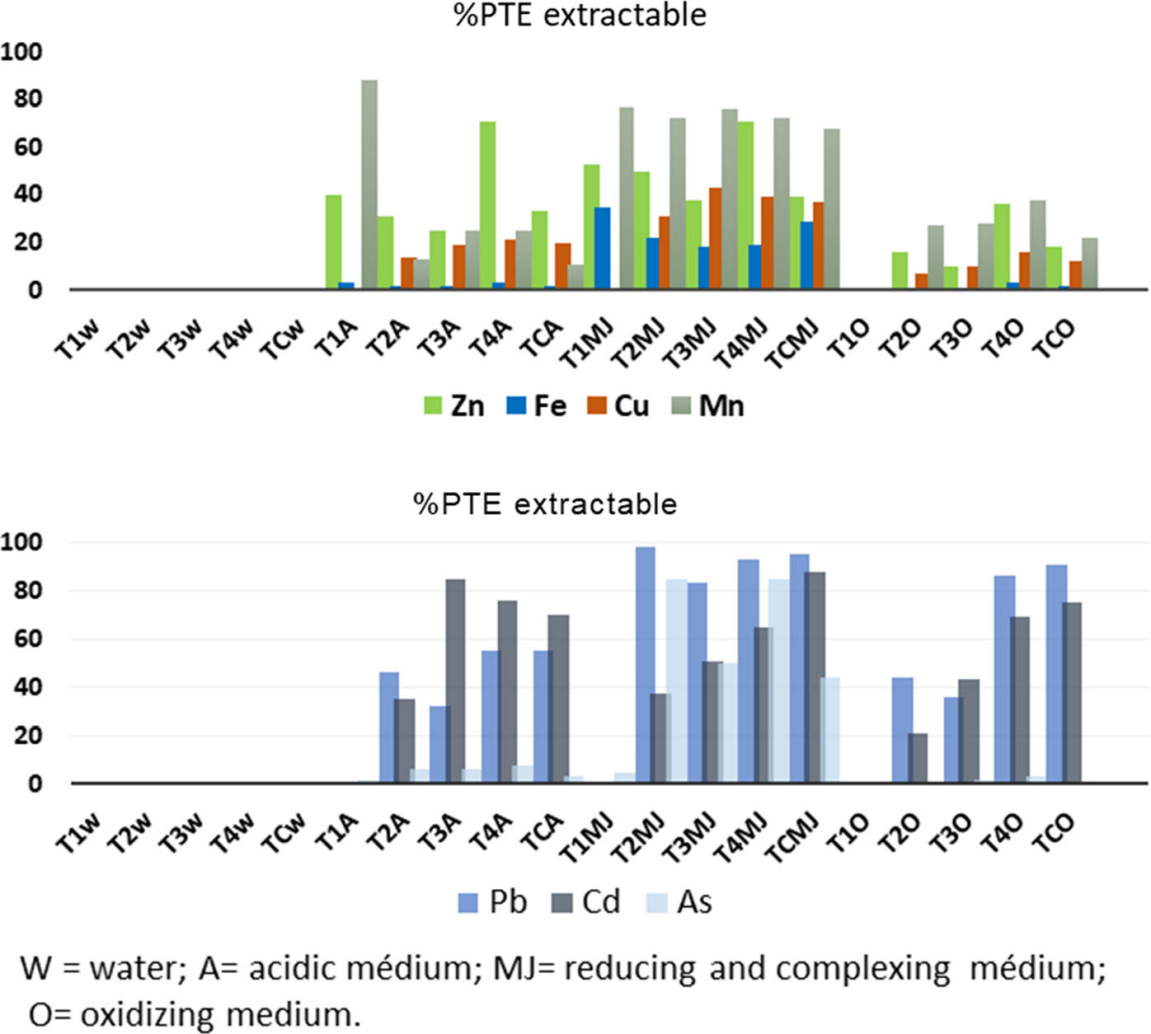
Results of the mobility study of PTEs in the Technosols (T1, T2, T3, T4 and TC) expressed in percentage of element extraction in the extractant medium referred to the total concentration of the element (W = water; A = acidic medium; MJ = complexing and reducing medium; O = oxidizing medium)

The results presented in Fig. 2 show the extraction percentages with respect to the total PTE concentration of the analyzed soils. In general, the medium that mobilized less nutrients and PTEs was water (watering, raining) while the most mobilizing was the complexing–reducing medium (MJ extraction). Intermediate results were obtained for the media simulating acid and oxidizing environments.

Arsenic was the least mobile element, and could only be mobilized in reducing conditions. Such behavior agrees with the observations made in other studies carried out with soils and sediments from nearby mining areas (Martínez-Sánchez et al., 2011) and emphasizes the importance of performing such studies to avoid any possible negative consequences for human health. The fact that As can be mobilized under certain conditions points to its possible uptake by plants and the consequent risk to the ecosystem.

There was a very close relationship between PTE concentration and mineralogy, as well as with their behavior in different extracting environments. The matrix that provided the highest PTE levels was the mine soil (TC), since the agricultural soil, T1, and the amendments had very low PTE concentrations, although the presence of the different amendments in a given soil type would condition the mobility of each element. Therefore, there were significant differences in PTEs mobility, which was affected by the calcium carbonate content (active and equivalent) and the type of amendment. An analysis of comparison of means, ANOVA, (Table 5) was carried out, selecting the type of extractant as an independent factor, and no significant differences were observed in the results, except in the case of Pb and Cd, which were affected differently in the means tested (value *p* < 0.05). When the type of soil was selected as an independent factor, the results showed great differences, deducing that each extractant acted differently for each element according to the type of considered soil. The differences observed in the results obtained were justified by the mineralogical compositions present in each soil. The correlation of calcite and dolomite from the extracted and total PTE values in different environmental conditions was very significant. Negative correlations could be observed for most of them in all the media tested, except water, in which the low values found (< QL) did not permit any correlation to be established. In this sense, there were also very significant and positive correlations with akaganeite and jarosite (Pearson correlation 0.77–0.98), confirming the influence of materials of mining origin on the behavior of PTEs and the immobilizing capacity provided by carbonated amenders (Pearson correlation calcite and dolomite, negative value, 0.77–0.99). In addition, it should be noted that the incorporation of calcium carbonate in the soils resulted in an increase in soil pH, an improvement in the regulatory capacity of an acid or oxidizing environment and the competition between calcium and the PTEs to form chelates and other complexes, as has been seen to occur in reducing and complexing media (Pérez-Sirvent et al., 2018).

**Table 5:** Summary of one-factor ANOVA (Factor: A = type of extractant (acidic medium, reducing and complexing medium, oxidizing medium); B = type of Technosols (T1, T2, T3, T4 and TC)) of the metal(oid)s extracted in the mobilization study

	A		B	
	F	Sig	F	Sig
Zn	2.573	.081	3.684	.034
Pb	3.333	.038	2.487	.098
Fe	.570	.688	7.070	.003
Cd	5.482	.006	1.238	.329
Cu	1.663	.211	5.281	.010
As	.687	.612	4.661	.016
Mn	1.411	.278	3.775	.032

The high mobilization that occurs in a reducing and complexing medium was due to the non-crystalline Fe oxyhydroxide (akaganeite) content, short-range-order mineral phases that are easily mobilized because of their low crystallinity. In such conditions, the behavior of the As associated with Fe follows the same pattern, which explains why this environmental condition induces the greater degree of mobilization (Martínez-López et al., 2019).

**Fig. 3:**
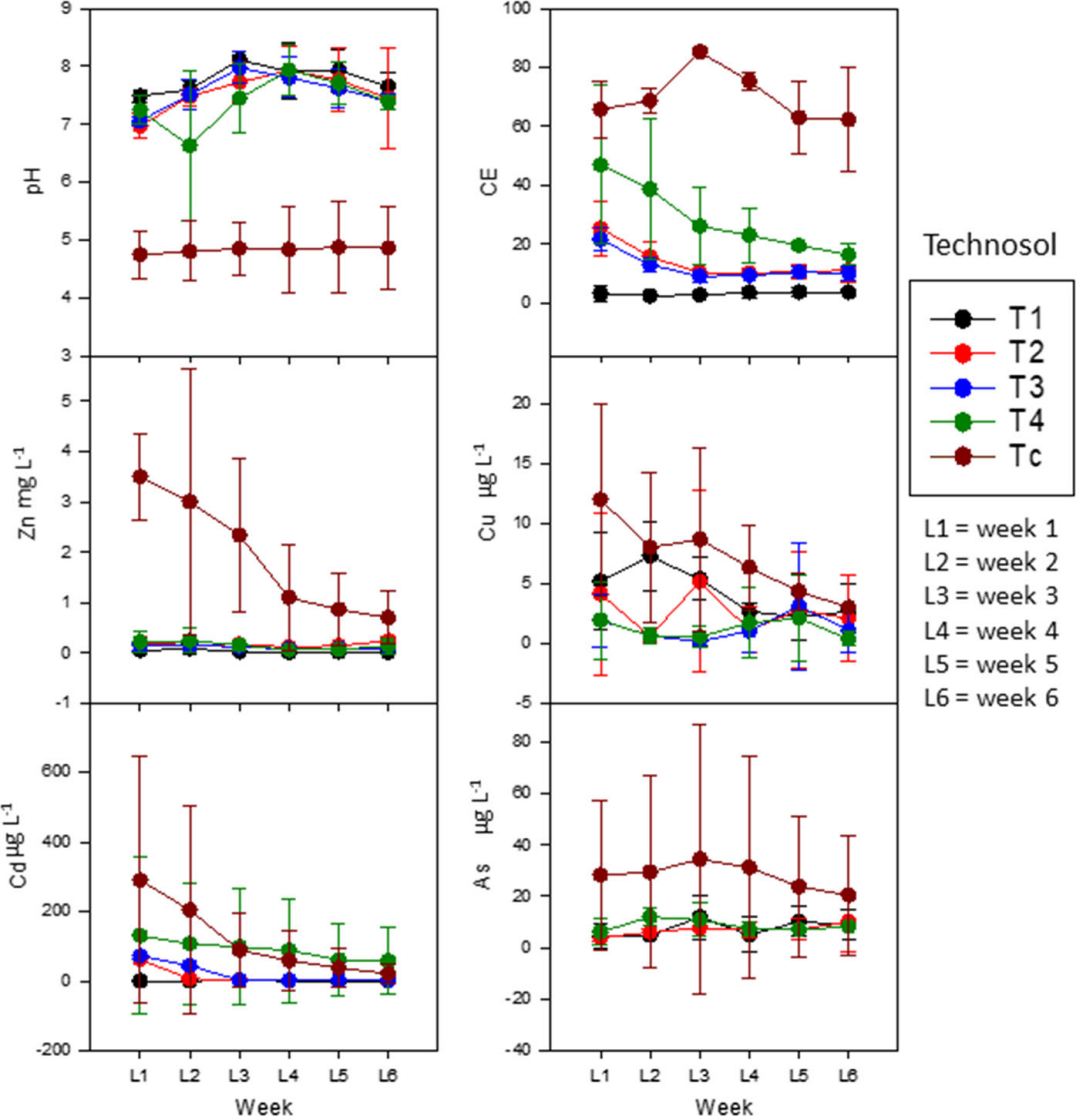
Temporal evolution of parameters in the leachates obtained from Technosols (T1, T2, T3, T4 and TC) in the cultivation period

Figure 3 shows the variations in the concentration of PTEs, EC and pH of the leachates obtained during the period of plant development, the sampling times varying according to the plant species growth cycle: shorter for lettuce and longer for onion and broccoli.

The pH values were not significantly affected by plant type and showed a constant trend during the observed periods in both remediated and non-remediated soils (T1 and TC). Electrical conductivity tended to decrease with time for all the crops in all soils containing materials of mining origin. Only the T1 soil, farmland soil, showed no variations in leachate conductivity during the weeks of growth. In no case was this parameter affected by the species being grown.

The PTE concentrations determined in the leachates were very low in all cases with values that were often below the detection limits. This agrees with the values obtained in the analysis of water mobilization, but as can be seen (Fig. 3), some variations were associated with the type of crop and the type of soil.

The general trend was for the PTE concentration to decrease as the growing period progressed, either because of immobilization in the rhizosphere or because the easily mobilized fraction was absorbed by the plant, thus decreasing the concentration in the leaching water. The sharpest decrease was normally evident in the third week, although the concentration of some PTEs increased after this time, which would have been favored by the root growth of plants during the vegetative period (Jungk & Claassen., 1997; Wang et al., 2016). The greatest decreases were those of micronutrients, such as Zn and Cu, while those showing variations and a less pronounced decrease were Cd and As. The Pb concentration could not be evaluated because in all cases it was below the detection limit, while Fe and Mn behaved in a very similar way to Cu.

As regards the different plant species are concerned, the concentration of all the studied elements showed a clear tendency to decrease in lettuce and broccoli, while As and Cd tended to increase slightly in onion. Any variation regarding soil type was not significant, and very similar values were obtained for all of them, although they were slightly higher in TC.

### Absorption of PTEs by plants.

**Fig. 4:**
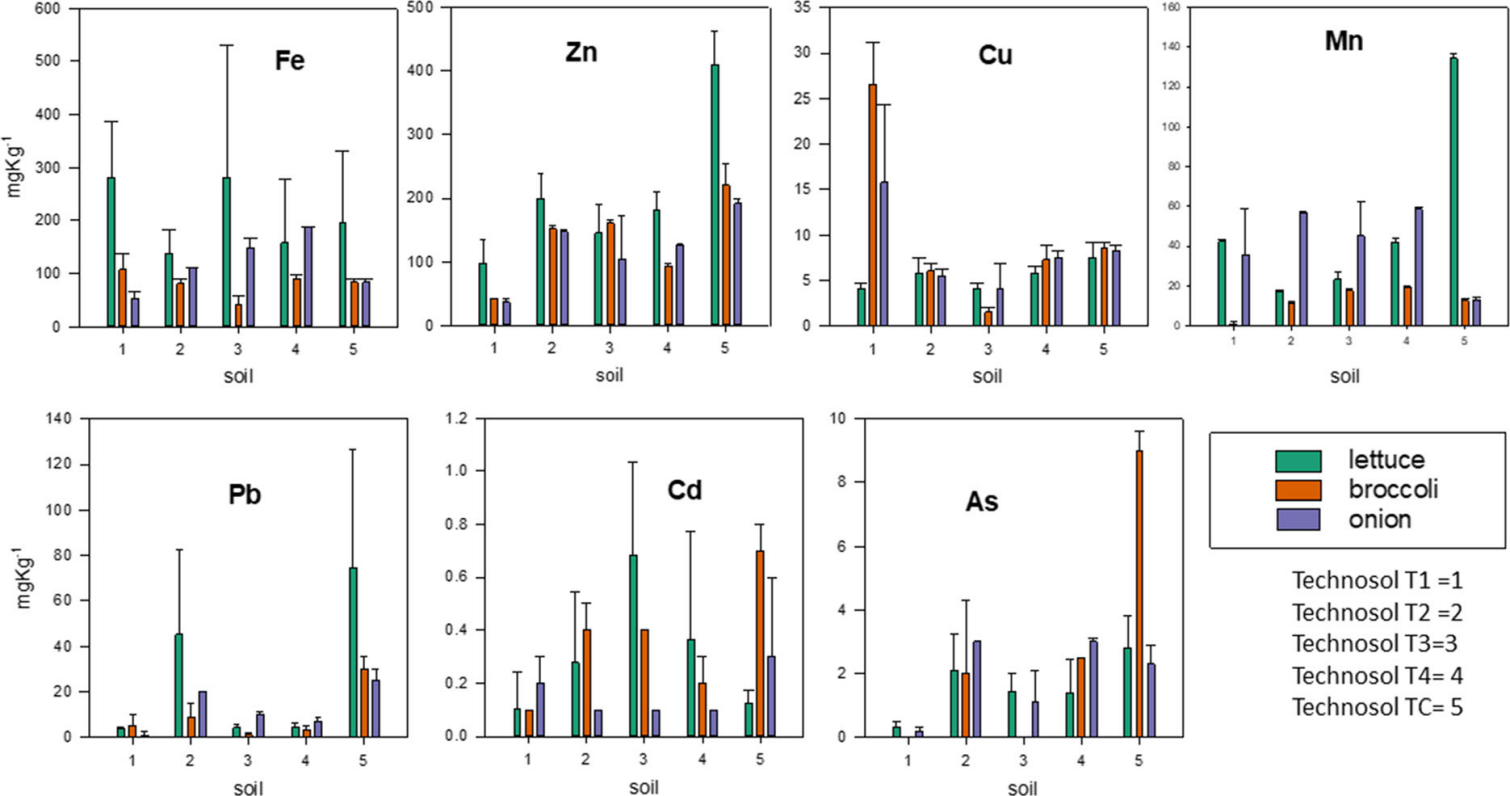
Levels of PTEs (median ± standard deviation) in the edible plants studied

Since the PTE concentration in the soils was very high, with the exception of T1, it could be assumed that phytotoxic effects for plant development should exist (Chaney, 1989). However, such effect was not evident in the plants tested, except for those grown in TC, in which less than 20% of the plants survived and yields were very low. It is interesting to note that the values obtained for PTE concentration in the edible part of plants grown in T1, T2, T3 and T4 soils were similar to the values reported in the literature for other edible plants (Kabata-Pendias and Mukherjee, 2007). On the contrary, for the plants grown in the TC soil, the values were above those that might be considered normal (Fig. 4).

Currently, in the European Community, there are only limits in Cd, Pb and Hg for fresh vegetable foods, and recommendations for As that do not include vegetables (COMMISSION REGULATION (EC) No 1881/2006). Only values higher than 0.10 mg kg^-1^ (fresh matter) in Pb and Cd are considered not recommendable for consumption. (Hg is not evaluated in this work.) According to this consideration, once the pertinent calculations from fresh matter to dried matter have been made, it is of note that only in TC and in some cases of T2 lettuce these limits were exceeded.

**Fig. 5:**
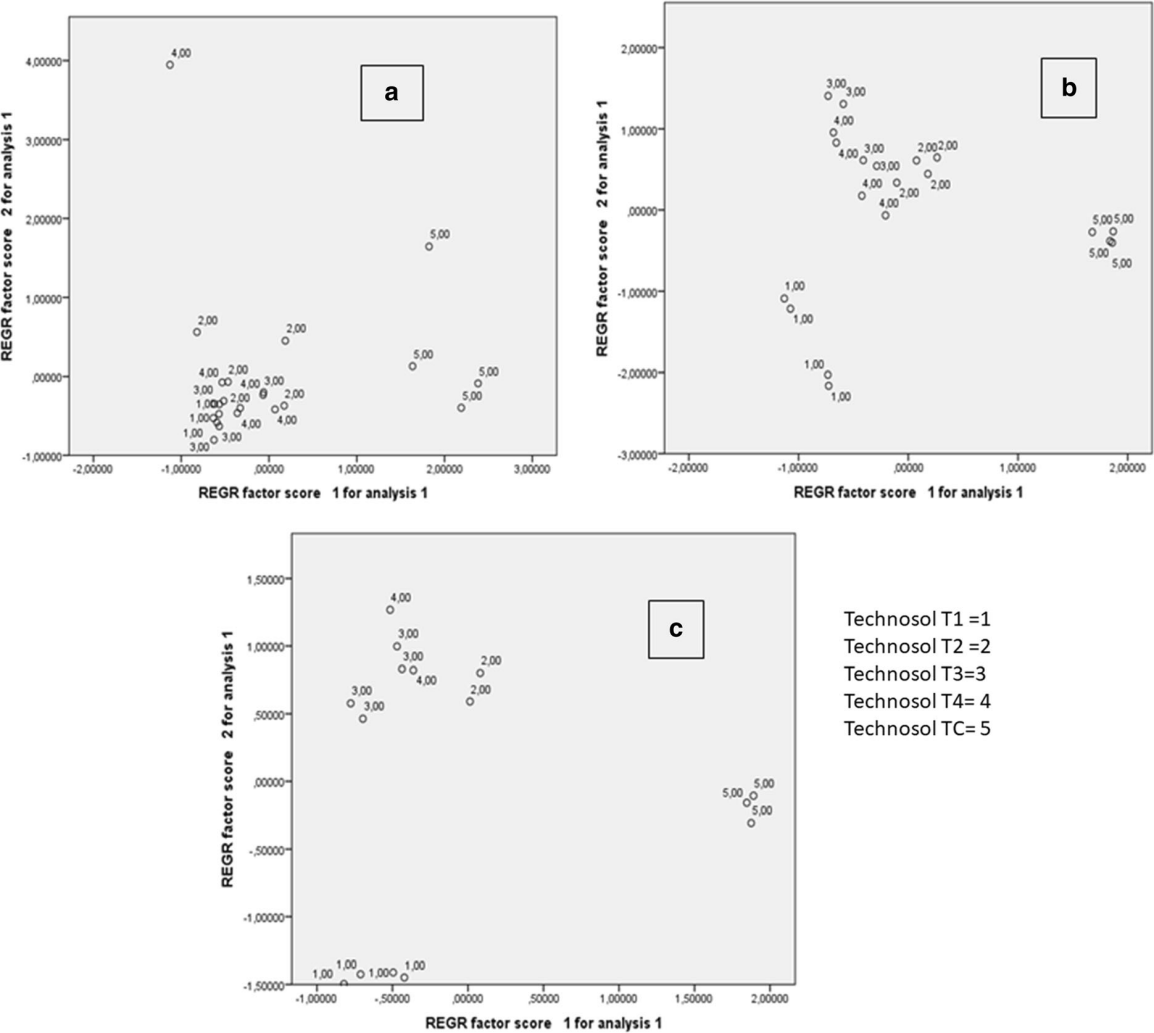
Graphical plots obtained in principal components analysis (F1 versus F2). a) Lettuce *(Lactuca sativa*), b) broccoli (*Brassica oleracea var. italica*), c) onion (*Allium cepa*)

In order to reduce the number of variables influencing the absorption of PTEs by the plants analyzed, and consequently to clarify the different behavior of each element, a multivariate statistical analysis was carried out to check the plant/rhizosphere interrelationships in the different Technosols. In the principal component analysis performed, three factors provided an explanation for the variance close to 80% using a varimax rotation. Factor 1 grouped components of the rhizosphere, Zn in the plant (aerial parts and root) and As, Fe and Mn in the root, while Factor 2 covered the rest of the PTEs contained in the aerial parts and roots, except for Fe, Cd and Cu which were included in Factor 3. The graphical representation of the samples based on Factors 1 and 2 (Figure 5) shows how samples were grouped according to the soil type and plant. The weight of Factor 1 affected plants grown in TC with greater intensity, particularly lettuce. For broccoli and onion, the samples are clearly separated into three groups, samples grown in TC, agricultural soil samples (T1) and the rest of them, which is not the case for lettuce.

The different concentrations of PTEs in the plants studied were a consequence of both the type of soil and the plant in question. When ANOVA was carried out with all the samples depending on the type of soil where they grow, only the variables Fe and Mn concentrations in edible plants presented a *p* value >0.05. Repeating the ANOVA for each plant, significant differences were found for Pb and Fe in edible plant Zn and As in root, for broccoli in Cu root and onion, Cd in edible plant and As in root, with p value >0.05.

**Fig. 6:**
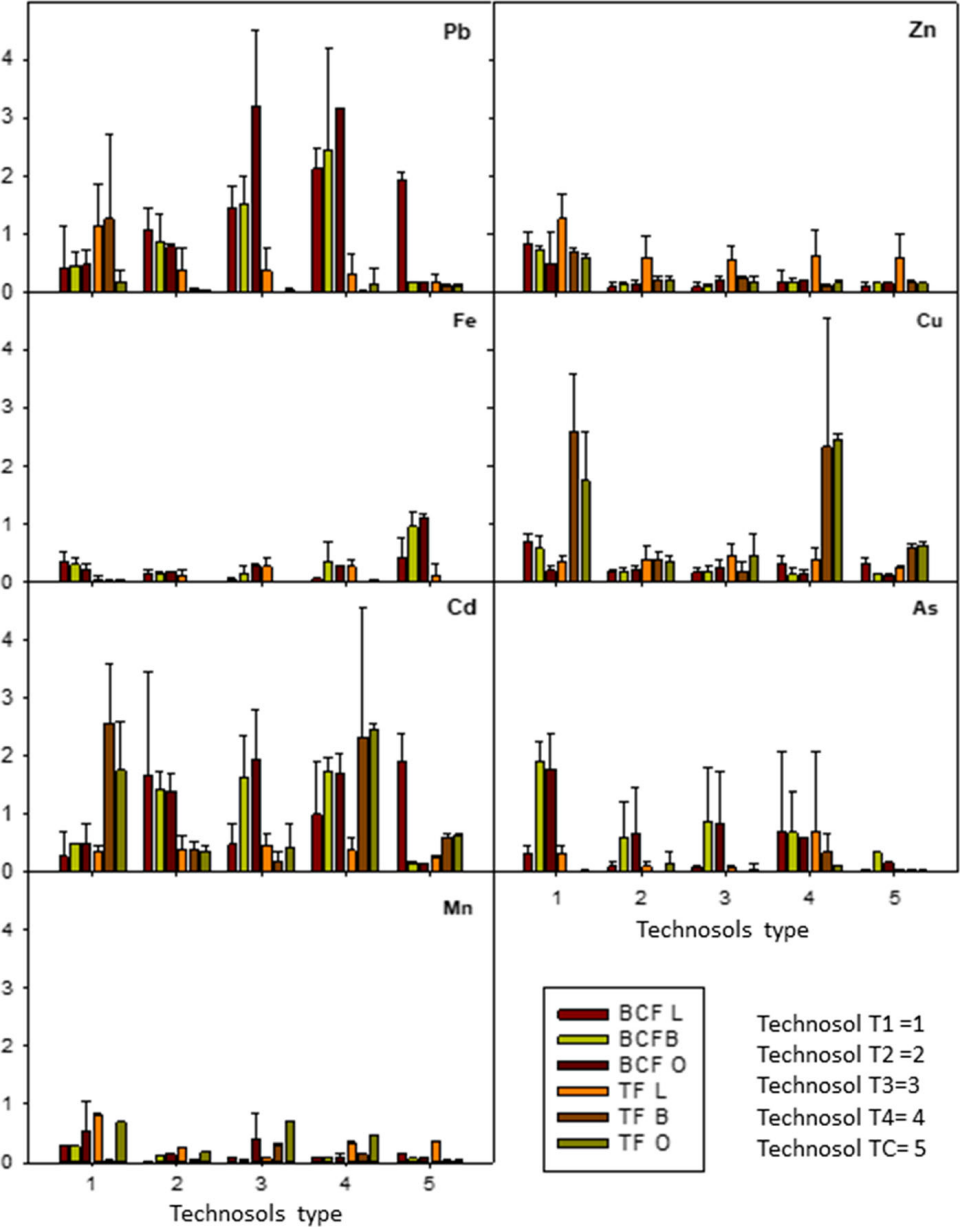
Bioconcentration factor (BCF) and transfer factor (TF) values (median ± standard deviation) for the different plants and Technosols

Figure 6 shows the mean values calculated for the transfer factor (TF) and bioconcentration factor (BCF) of the cultivated species.

In general, BCF and TF values were less than one, indicating that the plants did not tend to accumulate PTEs. On the contrary, it is of note that for the plants cultivated in TC, some values exceeded the unit. This should be taken into account when performing a risk analysis and also to discard certain plants as suitable for its cultivation in mining areas. Therefore, the remediated Technosols tested in these experiments did not present problems for the crops studied in this paper, making them suitable for the proposed objectives.

A one-way analysis of variance (ANOVA), selecting soil type as independent factor, and the calculated BCFs and TFs as variables showed significant variations with respect to the type of Technosol used (significance value of 0.000–0.008) for all the PTEs, with exception of Fe and As. When the analysis was repeated for each type of plant, the number of variables showing significant differences was higher for lettuce and onion (Table 6).

**Table 6:** Summary of one-factor ANOVA (Factor: carbonate calcium content) of BCF and TF obtained

	All samples		Lettuce		Broccoli		Onion	
	F	Sig	F	Sig	F	Sig	F	Sig
BCFZn	24.000	0.000	19.858	0.000	141.218	0.000	.790	0.557
BCFPb	6.151	0.000	1.181	0.352	4.656	0.012	6.003	0.010
BCFFe	17.802	0.000	5.085	0.006	10.231	0.000	100.240	0.000
BCFCu	9.276	0.000	17.313	0.000	9.001	0.001	0.645	0.643
BCFCd	3.842	0.008	2.231	0.106	14.590	0.000	7.023	0.006
BCFAs	3.798	0.009	0.820	0.529	3.974	0.022	2.544	0.105
BCFMn	4.402	0.004	1903.861	0.000	638.729	0.000	0.933	0.483
TFZn	6.491	0.000	2.843	0.055	123.752	0.000	18.848	0.000
TFPb	5.088	0.002	3.549	0.027	2.802	0.064	0.912	0.493
TFFe	1.223	0.312	2.688	0.065	5.794	0.005	10.300	0.001
TFCu	4.031	0.006	0.796	0.543	4.273	0.017	1.999	0.171
TFCd	4.031	0.006	0.796	0.543	4.273	0.017	1.999	0.171
TFAs	1.945	0.117	0.129	0.970	4.242	0.017	1.091	0.412
TFMn	4.002	0.007	695.815	0.000	224.052	0.000	3.249	0.059

## Summary of the main processes involved

**Fig. 7:**
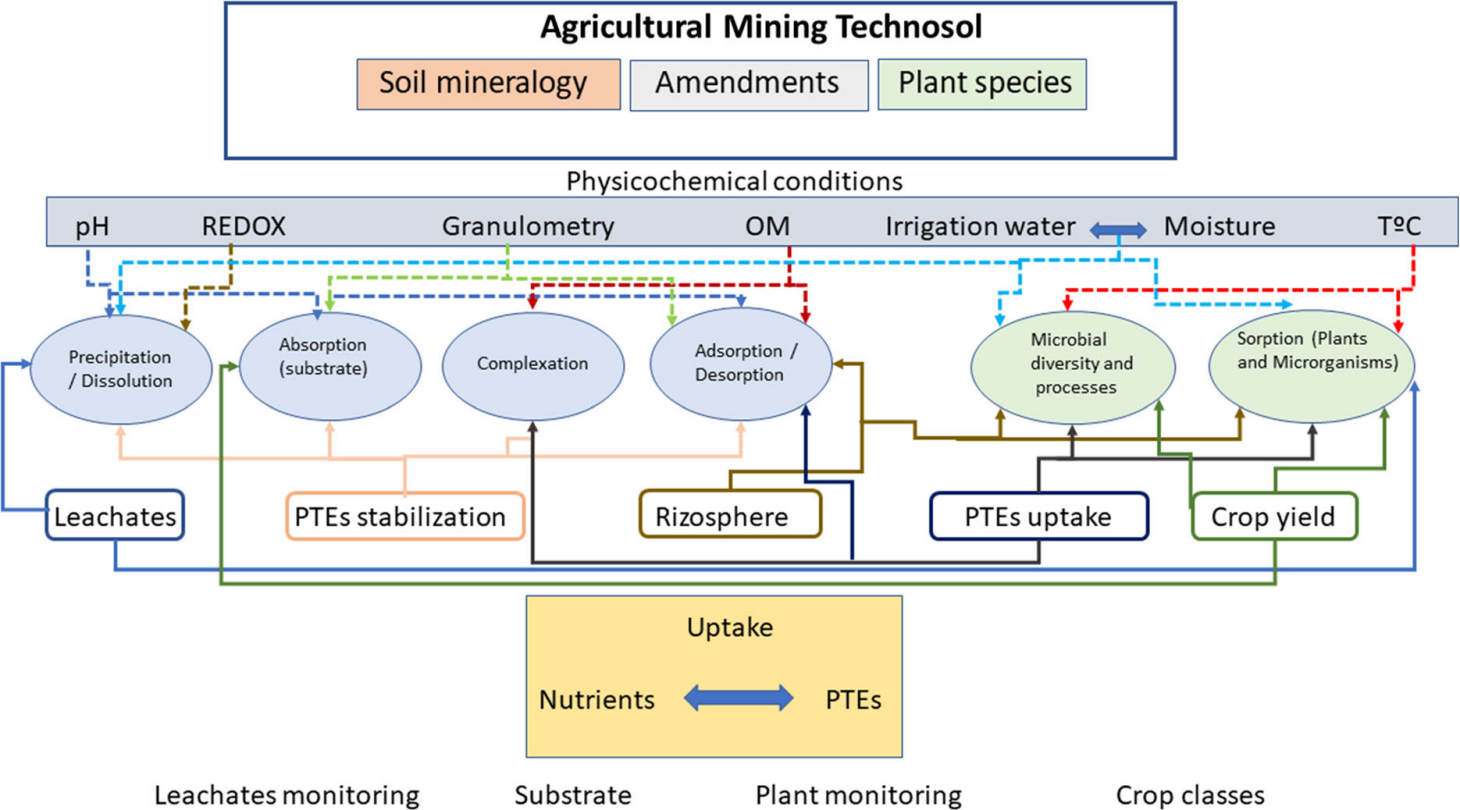
Diagram including the main physicochemical processes and conditions involved in the construction of a Technosol

Figure 7 summarizes the main hypotheses on which the experiment described in this paper is based: the design of the different proposed Technosols, the selected amendments and test plants, and the monitoring of the experiment in its different phases. The graph also considers the soil mineralogy conditions, all the reactions that may take place in the transfer balances between the rhizosphere and the plant and the physicochemical conditions (pH, EC, etc.). As a consequence, the composition and development of leachates, the stabilization of PTEs, the composition of the rhizospheres, the uptake of nutrients by plants and the vegetative development of crops are also affected.

The processes developed in each type of Technosol are related to their physicochemical properties, affecting the behavior of the plant’s rhizosphere. These chemical balances interact and can cause important effects on the uptake of PTEs by the plant. A general process can also be complicated by synergies and competitions between micronutrients and PTEs that may occur depending on environmental conditions (Palansooriya et al. 2020).

The presence of calcium carbonate affects the pH and particle size distribution of the soil, and therefore the mobility of the PTEs (precipitation reactions, absorption, adsorption, etc.) which is manifested in a decrease in the concentration of leachates, stabilization of the PTEs in the soil, low TF and high crop yields. On the other hand, there is a balance between micronutrients and PTEs in the plant uptake, producing competences and synergies for the uptake, depending in each case on the selected plant, which are manifested in the differences obtained in the plant concentrations according to the species and the type of soil.

## Conclusion

The study developed with three different plant species cultivated for human consumption (lettuce, broccoli and onion) in one uncontaminated soil and different Technosols allows the following conclusions: (1) The soils studied present limiting factors that prevent the normal development of crops and which are related to their pH and the salts and carbonates they contain. (2) The PTE levels found in the plant species studied are within the range of values mentioned in the literature when cultivated in recovered soils. However, when the plants grow in contaminated soils, the PTE levels vary greatly depending on the species being higher in onions than in lettuce. (3) Soil type, defined according to the carbonate content, determines the uptake by the plants of the trace elements considered in this work, through absorption by the roots and transport to the aerial/edible part. The type of plant also influences this process specially in the case of Fe, Cd and As.

The experiments made using LF or DCW for soil treatment allow obtaining crops that, in principle, do not present health risks and have a similar development to those grown in agricultural soil. The T2 soil that does not have amendments, in general, tends to behave like T3 and T4 (amended), although in the case of lettuce, it can give PTEs concentration values above those recommended.

Regarding the type of amendment, the differences between LF and DCW are not significant, so it is considered that both options can be valid for a remediation action of soils contaminated by PTEs. The monitoring of leachate and crops, as well as the selection of plant species, must be taken into account, among other aspects, for any project of soil recovery for agricultural use.
